# Evaluation of the efficacy of OLIF combined posterior internal fixation for single-segment lumbar tuberculosis: a single-center retrospective cohort study

**DOI:** 10.1186/s12893-022-01492-4

**Published:** 2022-02-13

**Authors:** Xing Du, Yunsheng Ou, Wei Luo, Guanyin Jiang, Wanyuan Qin, Yong Zhu

**Affiliations:** Department of Orthopedics, The First Affiliated Hospital of Chongqing Medical University, No.1 YouYi Road, Yuan Jia Gang, Yu Zhong District, Chongqing, 400016 China

**Keywords:** Lumbar tuberculosis, Oblique lumbar interbody fusion, Debridement, Internal fixation

## Abstract

**Objective:**

To evaluate the clinical efficacy of oblique lateral interbody fusion (OLIF) combined posterior fixation for single-segment lumbar tuberculosis (TB).

**Methods:**

The medical records of spinal TB patients who were admitted to our department from January 2016 to December 2018 were retrospectively reviewed, and those meeting the inclusion criteria were finally included for analysis. The operative time, operative blood loss, hospital stay, visual analogue scale (VAS) score, Oswestry disability index (ODI), Cobb angle of surgical segment, bone graft fusion rate, erythrocytic sedimentation rate (ESR), C-reactive protein (CRP), neurological function (ASIA grade) and complications of the included patients were all recorded and analyzed.

**Results:**

Thirty-nine patients with lumbar TB were finally included. The mean operative time, operative blood loss, and hospital stay were 135.8 ± 19.2 min, 239.4 ± 84.7 ml, and 9.5 ± 2.7 days, respectively. The mean follow-up time was 26.3 ± 7.5 months. During the follow-up, both VAS score and ODI were significantly improved at 1 month, 3 months, 6 months, 1 year postoperative, and the last follow-up, compared with preoperative (*P* < 0.001). Cobb angle was significantly corrected at 1 month postoperatively (*P* < 0.001), however, from 3 months postoperative to the last follow-up, Cobb angle was getting lost (*P* < 0.01). Bone graft fusion rate at 3 months, 6 months, 1 year postoperative, and last follow-up were 66.67%, 87.18%, 94.88%, and 100%, respectively. Compared with preoperative, ESR and CRP were both showed significant decrease at 1 and 6 months postoperative, and the last follow-up (*P* < 0.001). At the last follow-up, all patients had improvement in ASIA grade compared with preoperative (*P* < 0.001). Six patients were found with postoperative complications, and all were cured after active treatment.

**Conclusions:**

OLIF combined posterior internal fixation is safe and effective in the treatment of single-segment lumbar TB, with satisfactory pain relief, improvement of lumbar and neurological function, and deformity correction.

## Background

Osteoarticular tuberculosis (TB) is the most common form of extrapulmonary TB, with about 50% of cases occurring in the spine [1]. Lumbar TB is the most common spine TB, often resulting in vertebral destruction, collapse, spinal instability, deformity, and even neurological dysfunction in severe cases [2]. The treatment of spinal TB mainly includes anti-TB chemotherapy and surgery. It is currently considered that anti-TB chemotherapy is the cornerstone of spinal TB treatment and surgery is mainly used for patients with severe spinal instability, spinal deformity, or impaired neurological function [3].

At present, the surgical methods for lumbar TB include anterior approach surgery, posterior approach surgery, and combined anterior and posterior approach surgery, among which the posterior approach is the most common used [4]. Application of posterior approach surgery in lumbar TB has been showed satisfactory clinical efficacy and safety, including improvement in clinical symptoms, high bone fusion rate and good deformity correction effect [5]. However, cases of chronic back pain or lower limb weakness are not rarely reported after the posterior approach surgery which may be due to the stripping of unilateral or bilateral parvertebral muscles and the opening of the spinal canal during the surgical procedure [6, 7]. Thus, spinal surgeons have been trying to find more minimally invasive surgical methods for the treatment of lumbar TB.

Oblique lateral interbody fusion (OLIF) is a minimally invasive surgical technique, which has been widely used in the treatment of lumbar diseases in recent years. During the OLIF procedure, the surgeon enters the target intervertebral space through the anatomical gap between the anterior edge of the psoas major muscle and the abdominal aorta to perform discectomy, dural sac decompression, and also realize effective indirect decompression by placing a large cage [8]. OLIF technique has shown satisfactory clinical efficacy in the treatment of lumbar spinal stenosis and lumbar disc herniation [9, 10]. However, at present, few scholars have applied OLIF in the treatment of spinal TB. In our previous study, we found that OLIF combined posterior internal fixation in the treatment of degenerative lumbar spondylolisthesis has the advantages of less surgical invasion, better decompression effect and faster postoperative recovery (including back pain and lumbar function) compared with the posterior approach surgery [11]. Thus, we wanted to know whether these positive results of OLIF could be extended to patients with lumbar TB.

Therefore, we conducted this retrospective cohort study to evaluate the clinical efficacy of OLIF combined posterior internal fixation for single-segment lumbar TB.

## Method

Our present study was approved by the Ethics Committee of the First Affiliated Hospital of Chongqing Medical University (2017-067). Written informed consent was got from each included patient. This research was conducted in accordance with the Declaration of Helsinki (as revised in 2013) and reported in line with the STROBE criteria [12].

### Patients

The medical records of patients with spinal TB admitted to our department from January 2017 to June 2019 were retrospectively analyzed.

Inclusion criteria: (1) Age > 18 years; (2) Patients diagnosed with spinal TB by postoperative pathological examination; (3) Single-segment lumbar TB (L1/2–L4/5); (4) The operative method was one stage OLIF combined posterior internal fixation; (5) Follow-up duration > 12 months.

Exclusion criteria: (1) Previous spinal surgery history; (2) Patients with recurrent spinal TB after treatment; (3) Patients with incomplete medical records during perioperative or follow-up periods.

### Preoperative management

All patients underwent lumbar X-rays, CT, and MRI preoperatively to assess the extent of bone destruction and the size of the surgical window (the space between the anterior edge of the psoas major muscle and the abdominal aorta). Each patient received standardized oral anti-TB chemotherapy (isoniazid 300 mg/d, Rifampicin 450 mg/qd, pyrazinamide 1500 mg/qd, ethambutol 750 mg/qd) for 3–4 weeks before surgery. When erythrocyte sedimentation rate (ESR) dropped to normal level or was in the stage of significant decline, surgery could be performed. The patient's comorbidities, such as hypertension, diabetes, and hypoproteinemia, also need to be controlled at normal levels before the operation. For patients with paralysis or progressive neurological deterioration, surgery can be performed after 3–4 days of standardized anti-TB chemotherapy.

### Surgical procedure

After general anesthesia, the patient was placed in the lateral position with the severely damaged side of the vertebral body as the operative side. C-arm fluoroscopy was used to identify the surgical segments. Then, on the ventrolateral side, a 4 cm long incision parallel to the obliqus externus abdominis was made. The abdominal muscles were separated layer by layer until the extraperitoneal fat layer was seen. Carefully push the extraperitoneal fat forward with fingers and use the Cobb periosteum dissection device to locate the anterior edge of the psoas major muscle. Then, an S-shaped retractor was placed to protect the anterior vessel while the psoas major muscle was pushed backward with the Cobb periosteum dissection device and the OIF right-angle retractor was placed. The target intervertebral space was exposed between the vascular sheath and the psoas major muscle, a positioning needle was inserted, and the surgical segment was redefined by C-arm X-ray. Insert the dilator gradually and finally place the surgical channel (22 mm in diameter), and make sure there were no blood vessels or nerves in the channel. The surgical channel used in this operation was the OLIF 25 Access (Medtronic, USA). Then, TB abscesses, granulation tissue, dead bones, and necrotic intervertebral discs were completely removed, and the bone graft bed was also prepared. According to the size of the bone defect after debridement, a titanium mesh (IRENE, China) of appropriate size was selected, which was filled with the patient's autogenous iliac bone particles, and then implanted into the intervertebral space. A drainage tube was then placed and the surgical incision was closed layer by layer.

The patient was adjusted to the prone position, and a midline incision was made on the back of the waist to expose the surgical segments and adjacent vertebrae. The pedicle screws were implanted on both sides of one vertebrae above and below the surgical segments via the Witlse approach. When the pedicle on one side of the affected vertebrae was severely damaged, pedicle screw can be implanted on the contralateral side only. The C-arm X-ray was the used to confirm the screws were in good position. Finally, suture the surgical incision layer by layer.

### Postoperative management

When the postoperative drainage volume was less than 40 ml/d, the drainage tube was removed. Antibiotics were given prophylactively in the first 3 days after surgery, and lumbar spine X-ray was re-examined 3 days after surgery. Anti-TB chemotherapy was continued for 18–24 months after surgery. Lumbar spine X-ray, liver and kidney function were followed up at 1, 3, 6 and 12 months after surgery, lumbar CT and MRI were also re-examined when necessary.

### Outcomes

Clinical outcomes: (1) operative time, operative blood loss, and hospital stay; (2) visual analogue scale (VAS) score and Oswestry disability index (ODI); (3) neurological function (ASIA grade) and postoperative complications. (4) Erythrocyte sedimentation rate (ESR) and C-reactive protein (CRP).

Imaging outcomes: (1) surgical segment Cobb angle: the angle between the upper endplate of the upper vertebrae and the lower endplate of the lower vertebrae of the surgical segment. (2) Bone graft fusion: fusion was evaluated according to the criteria reported by Bridwell based on the lumbar spine CT findings [13]. In this study, Grade I and II of the Bridwell scale were defined as achieving bone graft fusion.

### Statistical analysis

Quantitative data were expressed as mean ± standard deviation (SD) and compared by paired *t* test. The Mann–Whitney rank sum test and the *χ*^2^ test were used to compare the ordered and disordered qualitative data, respectively. SPSS 19.0 was used for statistical analysis, and *P* < 0.05 was considered statistically significant. GraphPad Prism 6.0 was used for plotting.

## Results

**Fig. 1:**
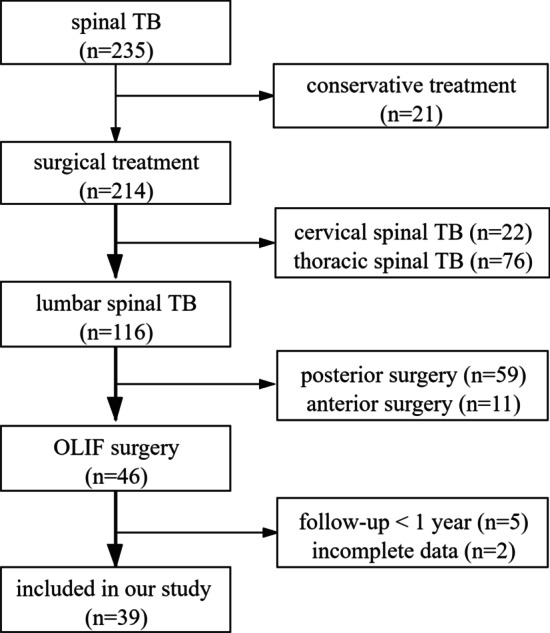
Flow chart of the inclusion and exclusion of patients

A total of 39 patients were finally included in this study (Fig. 1), including 17 males and 22 females. Preoperative American Standards Association (ASA) classification showed that 25 cases were Grade I, 12 cases were Grade II, and 2 cases were Grade II. The mean age, body mass index (BMI), operative time, operative blood loss, and hospital stay were 44.3 ± 11.2 years, 21.3 ± 2.1 kg/m^2^, 135.8 ± 19.2 min, 239.4 ± 84.7 ml, and 9.5 ± 2.7 days, respectively (Table 1).

**Table 1:** Baseline data of the included patients

Items	Value
Number of patients (n)	39
Gender (n), *Male/female*	17/22
Age (year), *mean* ± *SD*	44.3 ± 11.2
BMI (kg/m^2^), *mean* ± *SD*	21.3 ± 2.1
ASA grade (n)	
I	25
II	12
III	2
Operative time (min), *mean* ± *SD*	135.8 ± 19.2
Operative blood loss (ml), *mean* ± *SD*	239.4 ± 84.7
Hospital stay (day), *mean* ± *SD*	9.5 ± 2.7
Follow-up time (month), *mean* ± *SD*	26.3 ± 7.5

**Fig. 2:**
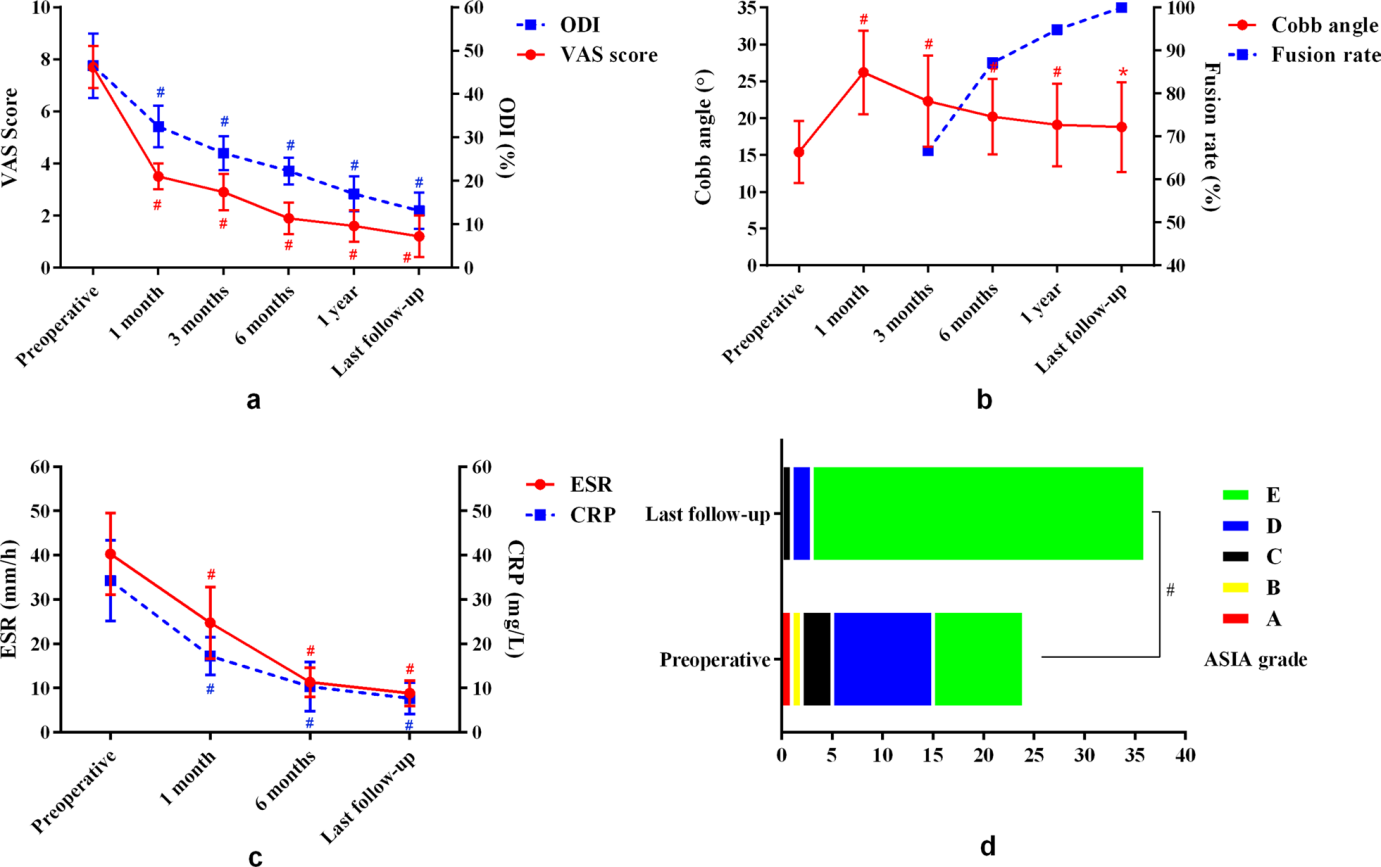
The changes of VAS score, ODI, Cobb angle, bone graft fusion rate, ESR, CRP, and ASA grade of the included patients during the follow-up. (^#^Compared with preoperative, *P* < 0.001; ^*^ Compared with preoperative, *P * < 0.01)

The mean follow-up time was 26.3 ± 7.5 months. Compared with preoperative, postoperative VAS score and ODI showed a trend of gradual decline (Fig. 2a). VAS score and ODI were significantly improved at all follow-up time points (1, 3, 6, 12 months, and the last follow-up) after surgery compared with those before surgery (all *P* < 0.001). Cobb angle was significantly corrected at 1 month postoperative (*P* < 0.001), however, from 1 month postoperative to the last follow-up, Cobb angle showed a trend of gradual loss (Fig. 2b). The correction and loss of Cobb angle were 11.6 ± 4.8° and 6.8 ± 2.2°, respectively. Bone graft fusion rate at 3 months, 6 months, 1 year postoperative, and the last follow-up were 66.67%, 87.18%, 94.88%, and 100%, respectively (Fig. 2b). ESR and CRP were both showed a trend of gradual decline after surgery compared with preoperative, postoperative ESR and CRP were significantly decreased at different follow-up time points (1, 6 months, and the last follow-up) (all *P* < 0.001) (Fig. 2c). Compared with preoperative, the ASIA grade at the last follow-up of all patients showed significant improvement (*Z* = − 3.578, *P* < 0.001), with 1 case of Grade A, 1 case of Grade B, 3 cases of Grade C, 10 cases of Grade D, and 24 cases of Grade E before surgery, and 1 case of Grade C, 2 cases of Grade D, and 36 cases of Grade E at the last follow-up (Fig. 2d).

Six patients were found with postoperative complications with the rate of 15.38%, including 1 case of neurological injury, 1 case of vascular injury, 1 case of instrument failure, a1 case of incision infection, and 2 cases of lower limb weakness and numbness (Table 2). All complications were cured after active treatment.

**Table 2:** Postoperative complications of the included patients

Complications	Value
Neurological injury	1 (2.56%)
Vascular injury	1 (2.56%)
Instrument failure	1 (2.56%)
Incision infection	1 (2.56%)
Lower limb weakness and numbness	2 (5.13%)
Total	6 (15.38%)

**Fig. 3:**
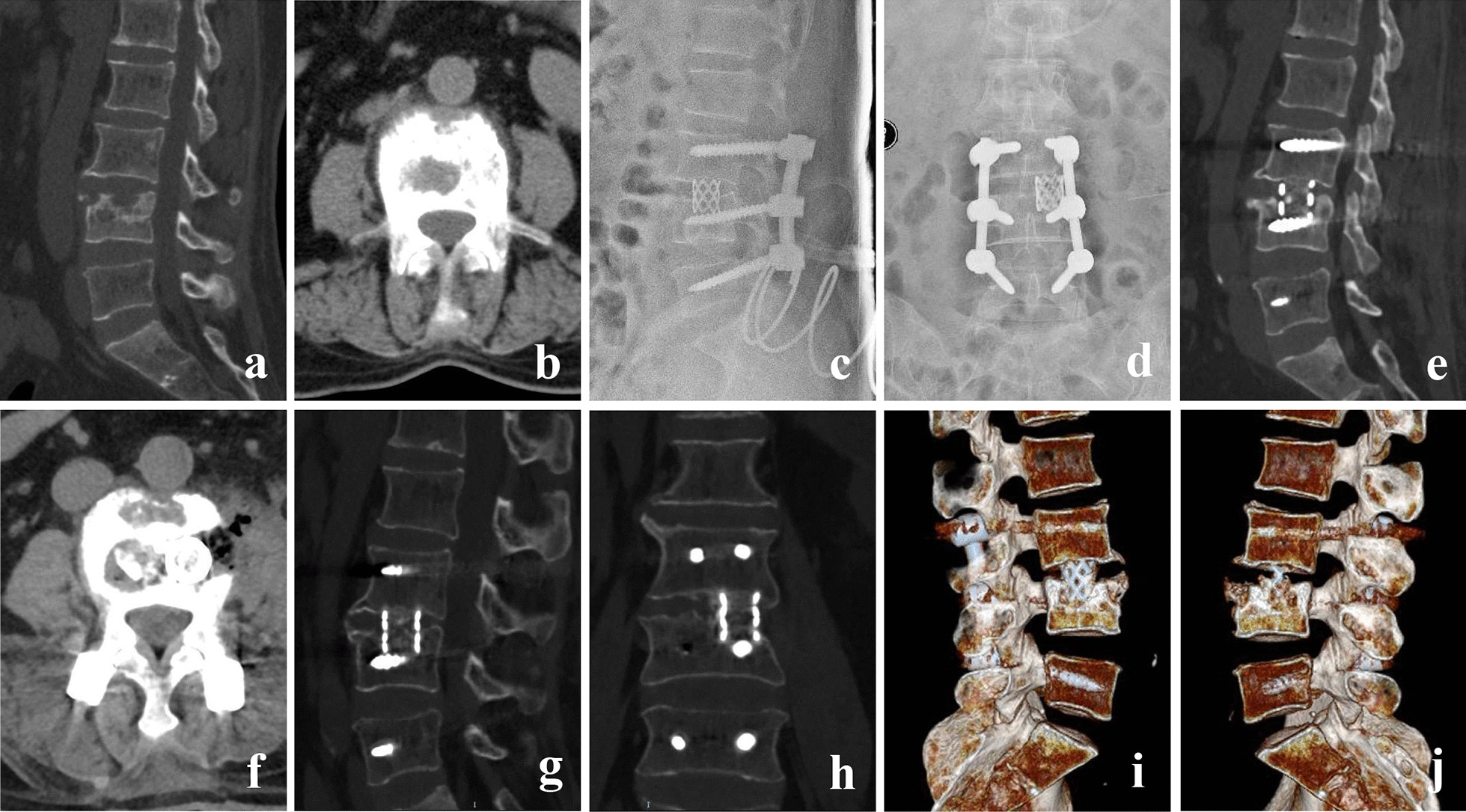
A 64-year-old female with L3-4 TB. (**a**, **b**) Preoperative CT showed that L3 and L4 vertebra body and the intervertebral disc were destroyed. (**c**, **d**) Postoperative X-ray. (**e**, **f**) CT at 3 months postoperative showed good location of titanium cage and posterior instrument. (**g**, **h**) CT at 6 months postoperative showed bone fusion between L3 and L4. (**i**, **j**) 3D-CT at 18 months postoperative showed good location of titanium cage and posterior instrument

**Fig. 4:**
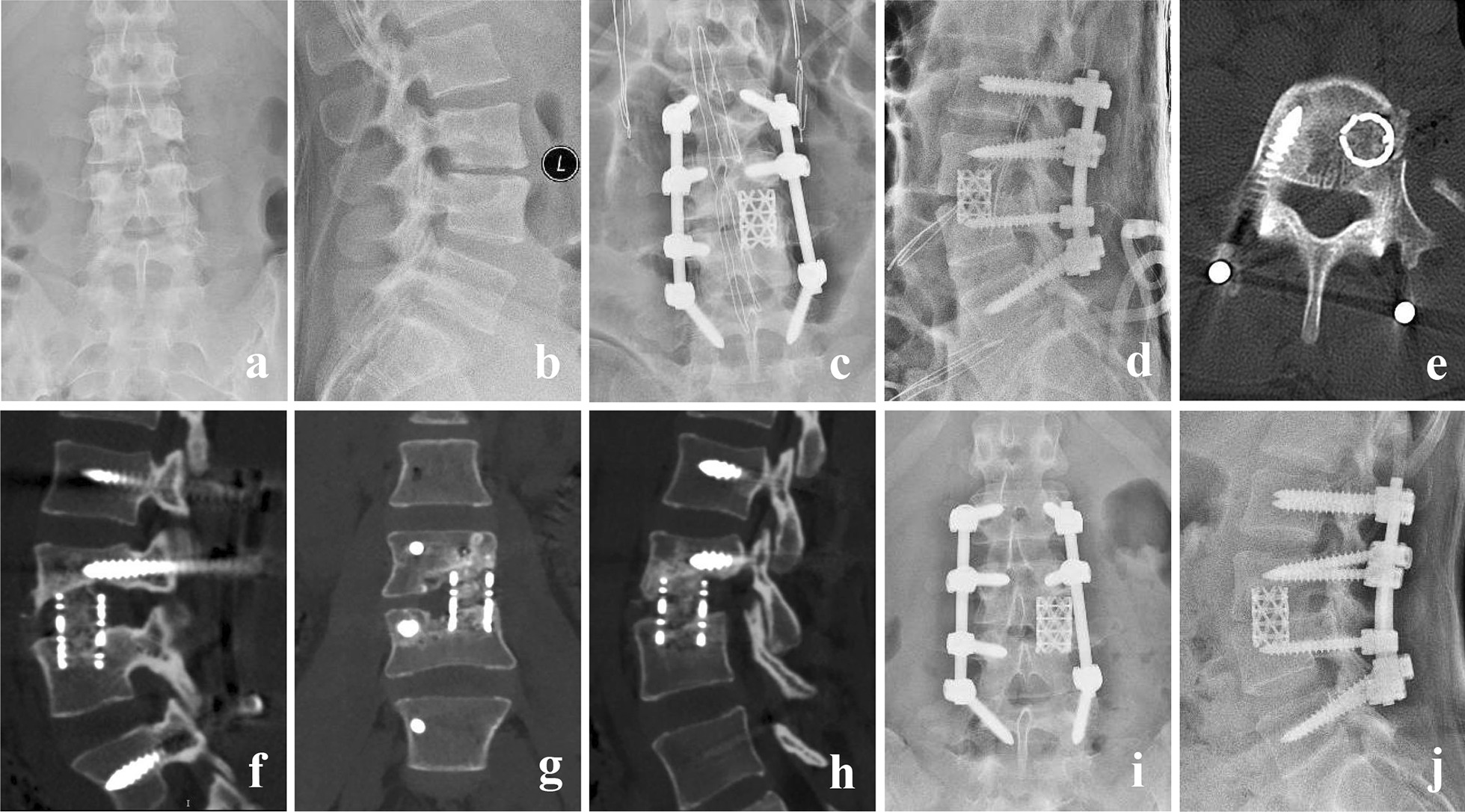
A 28-year-old female with L3-4 TB. (**a**, **b**) Preoperative X-ray showed that the vertebral gap between L3 and L4 was narrow, and vertebra body was partly destroyed. (**c**, **d**) Postoperative X-ray. (**e**, **f**) CT at 3 months postoperative showed good location of titanium cage and posterior instrument. (**g**, **h**) CT at 6 months postoperative showed bone fusion between L3 and L4. (**i**, **j**) X-ray at 14 months postoperative showed good location of titanium cage and posterior instrument

*Typical cases* Figs. 3 and 4.

## Discussion

In this study, all lumbar TB patients underwent OLIF combined posterior internal fixation, which was similar to traditional combined anterior and posterior spinal TB surgery. However, we did not find large surgical trauma in this study. On the contrary, the operative time and operative blood loss reported in this study were both lower than those of only posterior approach surgery for lumbar TB as we previously reported [14]. We thought there may be the following reasons: (1) During OLIF surgery, the surgeon could reach the lesion segment and perform the procedures such as TB lesion debridement, bone grafting and fusion under the direct vision [15]; however, in the traditional posterior approach surgery, these above procedures were all conducted in a blind field of vision especially the opposite side of the TB lesion [16]. (2) In OLIF combined posterior internal fixation surgery, the surgeon did not need to open the spinal canal [17]; but in posterior surgery, the surgeon need to remove at least one side of the lamina and facet articular process to create a window for operations such as debridement of TB lesions [18]. (3) In our present study, posterior pedicle screw internal fixation instrument was inserted via the bilateral Witlse approach, the surgeon did not need to dissection the posterior paravertebral muscles; but in traditional posterior approach surgery, at least one side of paravertebral muscles should be completely dissected from the lamina [19]. Moreover, this study also found that lumbar TB patients treated with OLIF combined posterior internal fixation had a significantly shorter hospital stay than those patients treated by posterior surgery as we previously reported. This may be related to the smaller surgical trauma of the surgical method in our present study.

In our present study, VAS score and ODI both showed significant improvement at each follow-up time points postoperatively compared with those before surgery. This result confirmed that OLIF combined posterior internal fixation in the treatment of lumbar TB was effective in alleviating clinical symptoms. Possible reasons were as follows: (1) Radical debridement and effective anti-TB chemotherapy were helpful to TB control and thus beneficial to pain relief and lumbar function recovery [20]. (2) Paravertebral muscle was an important factor affecting low back pain and lumbar function [21]. In OLIF combined posterior internal fixation surgery, surgeon inserted pedicle screws via the bilateral Witlse approach, and did not strip the paravertebral muscles [17]. (3) Implantation of the titanium cage and pedicle screw restored the height and stability of the diseased segments.

During the follow-up, a high and satisfactory bone graft fusion rate was also found. In this study, we used a titanium cage filled with granular bone for anterior column reconstruction, and our previous study had confirmed that the fusion time of granular bone graft was shorter [14, 18]. Compared with preoperative, Cobb angle was significantly corrected at 1 month postoperative, and the correction ability was larger than that reported in the posterior approach surgery. This maybe due to the following reasons: (1) In the OLIF procedure, a large titanium cage with a certain inclination angle can be placed in the diseased segment, which facilitates the reconstruction of the anterior column, correction of the kyphosis deformity, and recovery of the lordosis of the lumbar spine [22, 23]. (2) In this study, we combined the posterior pedicle screw internal fixation on the basis of OLIF, which was beneficial to the correction and maintenance of Cobb angle [24]. During the follow-up, Cobb angle showed a trend of gradual loss, but the loss was similar to that reported in posterior approach surgery. The possible reasons were as follows: (1) In this study, titanium cage was used to reconstruct the anterior column. Titanium cage had large elastic modulus and sharp edge, and thus was easy to subside [25]. (2) Posterior fixation system could not only help maintain Cobb angle correction but also reduce its loss. (3) Wearing a lumbar brace for 3 months after surgery was also conducive to reducing the Cobb angle loss [26]. The Cobb angle loss was related to the bone graft fusion condition. In this study, most patients (87.18%) had achieved bone graft fusion at 6 months, and small Cobb angle loss and significant VAS score and ODI improvement were also found at 6 months postoperatively. This result confirmed our previous findings that once the bone graft fusion was achieved, the Cobb angle loss was slight and did not affect the relief of patient's clinical symptoms [27].

The ESR, CRP and ASIA grade (neurological function) were all showed significant decrease during the follow-up. This was mainly related to the standardized anti-TB chemotherapy, radical debridement of TB lesions, effective spinal canal decompression, anterior column reconstruction, and spinal stability maintenance [28]. It was reported that segmental artery injury, transient thigh numbness were the common complications of OLIF surgery [29], because the lumbar plexus, lumbar sympathetic trunk and segmental artery are all located laterally in front of the lumbar vertebrae and susceptible to being irritated or injured. In this study, we found the complication rate was 15.38% which was similar to previous studies. All complications were cured after active treatment, this also indicated the safety of OLIF combined posterior internal fixation surgery for lumbar TB.

In our opinion, the operative indications of OLIF combined posterior internal fixation for lumbar TB were as follows: (1) Severe back pain, conservative treatment was ineffective or had poor effect; (2) Progressive neurological impairment or paralysis; (3) Progressive-aggravated spinal instability or severe deformity; (4) Single-segment lumbar TB (L2–L5); (5) TB lesion i\was mainly located in the anterior column of the vertebral body, with little involvement in the posterior column; (6) Preoperative CT or MRI showed an appropriate surgical window between the psoas major muscle and the abdominal aorta [30].

Based on our preliminary experience, in the treatment of lumbar tuberculosis with OLIF combined posterior internal fixation, we recommend the followings: (1) During the debridement of TB lesion via the lateral anterior approach, the sclerotic bone and the bone bridge between the anterior edge of the vertebral bodies should be preserved as much as possible. (2) The posterior internal fixation should be performed via the bilateral Witlse approach or by the percutaneous pedicle screw fixation to reduce the iatrogenic injury to the posterior paravertebral muscle and ligament complex. (3) Posterior rigid fixation with minimal or no intervertebral distraction.

The present study also has several limitations. First, it was a single-center retrospective study. Secondly, this study was a cohort study without setting a control group, so it was hardly to compare the efficacy of OLIF and other surgical methods. Third, the sample size was small and the follow-up time was short.

In conclusion, OLIF combined posterior internal fixation is safe and effective in the treatment of single-segment lumbar TB, with satisfactory pain relief, improvement of lumbar and neurological function, and deformity correction. However, due to the limitations of this study, the above conclusions need to be further validated by high-quality prospective controlled study with large sample and long follow-up.

## Data Availability

The datasets generated and analyzed during the current study are not publicly available due to patient privacy but are available from the corresponding author on reasonable request.

## Abbreviations

OLIF: Oblique lateral interbody fusion
TB: Lumbar tuberculosis
VAS: Visual analogue scale
ODI: Oswestry disability index
ESR: Erythrocytic sedimentation rate
CRP: C-reactive protein
SD: Standard deviation
ASA: American Standards Association
BMI: Body mass index
